# Peripersonal space around the upper and the lower limbs

**DOI:** 10.1007/s00221-022-06387-7

**Published:** 2022-06-21

**Authors:** Elena Gherri, Aolong Xu, Elisabetta Ambron, Anna Sedda

**Affiliations:** 1 Department of Philosophy and Communication, University of Bologna, Via Azzo Gardino 23, 40122 Bologna, Italy; 2 Human Cognitive Neuroscience, University of Edinburgh, Edinburgh, UK; 3 Laboratory for Cognition and Neural Stimulation, Neurology Department, School of Medicine, University of Pennsylvania, Philadelphia, PA USA; 4 Department of Psychology, Heriot-Watt University, Edinburgh, UK

**Keywords:** Multisensory integration, Peripersonal space, Crossmodal congruency task, Foot representation, Hand representation

## Abstract

Peripersonal space (PPS), the space closely surrounding the body, is typically characterised by enhanced multisensory integration. Neurophysiological and behavioural studies have consistently shown stronger visuo-tactile integration when a visual stimulus is presented close to the tactually stimulate body part in near space (within PPS) than in far space. However, in the majority of these studies, tactile stimuli were delivered to the upper limbs, torso and face. Therefore, it is not known whether the space surrounding the lower limbs is characterised by similar multisensory properties. To address this question, we asked participants to complete two versions of the classic visuo-tactile crossmodal congruency task in which they had to perform speeded elevation judgements of tactile stimuli presented to the dorsum of the hand and foot while a simultaneous visual distractor was presented at spatially congruent or incongruent locations either in near or far space. In line with existing evidence, when the tactile target was presented to the hand, the size of the crossmodal congruency effect (CCE) decreased in far as compared to near space, suggesting stronger visuo-tactile multisensory integration within PPS. In contrast, when the tactile target was presented to the foot, the CCE decreased for visual distractors in near than far space. These findings show systematic differences between the representation of PPS around upper and lower limbs, suggesting that the multisensory properties of the different body part-centred representations of PPS are likely to depend on the potential actions performed by the different body parts.

## Introduction

Peripersonal space (PPS) is defined as the space immediately surrounding the bodies of primates and humans (Clery et al. 2015; Pellegrino and Ladavas 2015; Serino 2019). This sector of space is extremely relevant functionally, as this is the space where interactions between the individual and its environment occur. For example, PPS is crucial when individuals need to defend themselves from approaching threats (e.g. Graziano and Cooke 2006; Sambo and Iannetti 2013) but also when they interact with objects and other individuals (e.g. Brozzoli et al. 2010; Làdavas and Serino 2008).

The representation of PPS in the brain is mediated by dedicated brain circuits characterized by specific multisensory properties (e.g. Graziano and Gross 1993; Graziano and Cooke 2006; Rizzolatti et al. 1981). Neurophysiological studies in primates have identified a neural population of visuo-tactile neurons in the ventral premotor cortex whose visual receptive fields (RFs) are anchored to tactile ones (e.g. Graziano et al. 1994; Rizzolatti et al. 1997). These neurons show strong responses to visual stimuli but only when these are presented close (within 10–20 cm) to the body part to which their tactile RFs are anchored. Because these neurons integrate tactile information from the body and visual information from the space surrounding it they are considered the neural substrate of PPS coding in the brain (e.g. Gross and Graziano 1995).

Converging evidence from neuropsychological and behavioural studies have shown that a similar system responsible for PPS encoding is present also in humans and is characterized by analogous multisensory properties (e.g. Grivaz et al., 2017). One well-established behavioural paradigm used to investigate the multisensory properties of PPS is the crossmodal congruency task (CCT) (Maravita et al. 2002; Pavani et al. 2000; Spence et al. 2004a, b). In this classic task, participants are instructed to hold two foam cubes, one with each hand, using their thumb and index fingers. On each trial, one task-relevant tactile target (a vibration) and one task-irrelevant visual distractor (a flashing light) is presented to one of four possible locations randomly (top or bottom of the cube, either to the same hand or the opposite hand). Participants are instructed to determine the elevation (top vs. bottom) of the tactile target while ignoring the visual distractor. Typically, responses are slower and less accurate when the distractor is presented at an incongruent elevation with respect to the target (e.g., bottom target and top distractor), compared to congruent elevations (e.g. top target and top distractor). The size of this crossmodal congruency effect (CCE), calculated by subtracting the reaction times (RTs) measured on congruent trials from those on incongruent trials, decreases as the visual distractor is presented further away from the tactile target (e.g. Maravita et al. 2002; Spence et al. 2004a, b). For example, the size of the CCE is typically reduced when the visual distractor is presented close to the hand that did not receive the tactile target (e.g. Pavani et al. 2000; Spence et al. 2004a, b). The dependence of the CCE on the target-distractor distance was also shown in studies in which participants performed the CCT with the two hands close together vs. far apart (5 vs. 100 cm; e.g. Soto-Faraco et al. 2004). Results showed larger CCEs when participants placed their hands closer than further apart. The spatial specificity of the CCE suggests that the underlying mechanism may be related to the visuo-tactile neurons discussed above.

The spatial properties of the hand PPS have been well characterised in a series of studies investigating PPS plasticity (e.g. Holmes and Spence 2004; Holmes et al. 2007, Holmes 2012). When the CCE was measured for visual distractors presented at three distances from the hands: near (directly next to the hand), middle (28 cm from the hands) and far (56 cm from the hands), reliable CCEs were observed at near and middle locations but not at far locations, suggesting that the boundary of hand PPS was located between the locations of middle and far distractors (Holmes 2012). Furthermore, the fact that the size of the CCE was reduced at middle as compared to near distractor locations suggests that the visuo-tactile integration which characterises PPS is maximal next to the hand and decreases steadily as visual stimuli are presented further away from the tactile target (e.g. Holmes 2012).

Although the original electrophysiological studies in monkeys have shown that neurons anchored to different body parts have different spatial properties, thus far the vast majority of behavioural studies in healthy participants have explored PPS around the upper limbs. Only recently researchers have started to investigate the presence and spatial properties of PPS around other body parts such as the face (e.g. Farne et al. 2005; Serino et al. 2015), torso (e.g. Alsmith and Longo 2014; Noel et al. 2015; Serino et al. 2015), and lower limbs (Pozeg et al. 2015; Scandola et al. 2016; Schicke et al. 2009; Stone et al. 2018; van Elk et al. 2013). The space around the lower limbs has been first investigated by Schicke et al. (2009) who adapted the CCT to investigate the presence of PPS around hands and feet. Results showed a reliable CCE in the feet task which was comparable to that observed in the hand task, suggesting a representation of PPS also around the lower limbs. Interestingly, in a follow-up experiment visual distractors presented near the feet also induced a CCE when the tactile targets were delivered to the hands and vice-versa. This indicate that different mechanisms other than the congruency of visuo-tactile RFs may be responsible for these effects.

Similarities between hand and feet PPSs were also reported in a later study (Van Elk et al. 2013) in which participants performed the CCT with their upper or lower limbs uncrossed or crossed. While in this study, the real limbs were hidden from view, rubber limbs were visible and positioned in anatomically congruent or incongruent postures with respect to the real limbs. Results in the uncrossed limbs position (for both the real and the rubber limbs) showed a similar CCE for the hands and feet suggesting similar PPS representations. However, the hand CCE but not the feet CCE was modulated when participants crossed their real limbs or when there was a postural incongruence between real and rubber limbs (Van Elk et al. 2013). These findings suggest that PPS representations for the hands but not for the feet are dynamically updated based on visual and proprioceptive cues, suggesting systematic differences between hand and feet PPS (Van Elk et al. 2013).

The evidence presented above suggests the presence of a PPS representation of the space around the feet. However, the properties of this spatial representation have been scarcely investigated thus far. To the best of our knowledge, only one study so far (Stone et al. 2018) has explored the question of the multisensory spatial properties of feet PPS by specifically assessing the multisensory properties of the space around the feet, presenting tactile stimuli directly to the feet, under static conditions (in the absence of real or induced feet movement). Stone et al. (2018) observed faster responses to tactile targets presented to the feet only when these were presented with an approaching visual distractor, not a receding one. They reported that PPS boundaries around the feet were located at approximately 70 cm from the feet. However, because only the feet PPS was tested in this study, it remains unclear how the spatial properties of foot PPS compare to those of hand PPS.

Upper and lower limbs are characterized by diverse functionals demands, as they allow to achieve different types of goal-directed actions. An intuitive example of these differences is thermosensitivity, which is twice in the hands than in the feet (Ackerley et al. 2014; Filingeri et al. 2018): hands are used by humans to reach out objects in the environment and these objects can be hot. More rarely, we need to be careful of hot obstacles located on the ground. Another example concerns the impact of posture on tactile localization. Preferential associations between body parts and spatial locations have been shown for the fingers (Romano et al. 2017). Participants are better able to localise a tactile stimulus when the thumb is in a relative bottom position while the middle finger is in a relative top position (Romano et al. 2017). However, this standard representation of body–space relationships was not observed for the toes (Manser-Smith et al. 2021). This difference between fingers and toes was attributed to the natural use of the limbs and the actions performed with these. Hence, functional differences between hand and feet make sense in evolutionary terms. Given the relevance of PPS for action purposes, hands and feet might be also characterized by different properties in terms of PPS.

To shed light on this question, we explored the spatial modulation of visuo-tactile integration around the upper and the lower limbs—as measured by the CCE induced by irrelevant distractors presented at near and far locations with respect to the tactually stimulated limb. In other words, we varied the distance in depth between the visual and tactile stimuli delivered to the hand and foot. Participants reported the elevation of a tactile target delivered to the dorsum of the right hand (in the hand task) or right foot (in the foot task), while ignoring a task-irrelevant visual distractor presented at a congruent or incongruent elevation. In different blocks of trials, the visual distractors were presented either right above the tactually stimulated limb (near distractor location), or further away from it (30–35 cm, far distractor location). Based on the existing literature (e.g. Holmes 2012), we expected to observe stronger CCEs in near than in the far space when the tactile target is presented to the hand, replicating the well-established result of stronger visuo-tactile multisensory integration in near as compared to far space. While a CCE is also expected to be found around the feet (c.f. Pozeg et al. 2015; Scandola et al. 2016; Schicke et al. 2009; Van Elk et al. 2013; Amemiya et al. 2019), there is no study to date that has directly measured CCE in the near and far space around the lower limbs, whereby the target-distractor distance is manipulated in depth (away from the body). Therefore, it is not clear whether the representation of PPS around the lower limbs is characterised by similar spatial properties as compared to that around the upper limbs. If this is the case, we expect similar patterns of CCE spatial modulations for hand and foot PPS, with stronger CCE for near than for far space.

## Methods

### Participants

Overall, 54 participants took part in this study. Two groups of participants performed different versions of the same task (using different effectors to respond, see explanation below). The first group included 34 participants. Two participants were excluded from the sample, because they were unable to perform the task above chance level (errors above 50%). Thus, the final sample for this group was of 32 participants [four male participants, mean age = 22.6 years, stand deviation (SD) = 1.30 years]. The second group included the remaining 20 participants [two male participants, mean age = 24.1 years, stand deviation (SD) = 1.41 years].

All participants were right-handed, according to the Edinburgh Handedness Inventory (Oldfield 1971).^1^ They were also naïve as to the purpose of the study, had normal or corrected to normal vision and were students at the University of Edinburgh or at the University of Glasgow in Scotland. The study was approved by the PPLS Research Ethics Committee of the University of Edinburgh and was carried out in accordance with the ethical standards as laid down in the 1964 Declaration of Helsinki and its later amendments. Participants gave written consent to participate in this study prior to the beginning of the study after the nature of the study had been explained to them. They were paid for their participation.

### Apparatus and materials

**Fig. 1:**
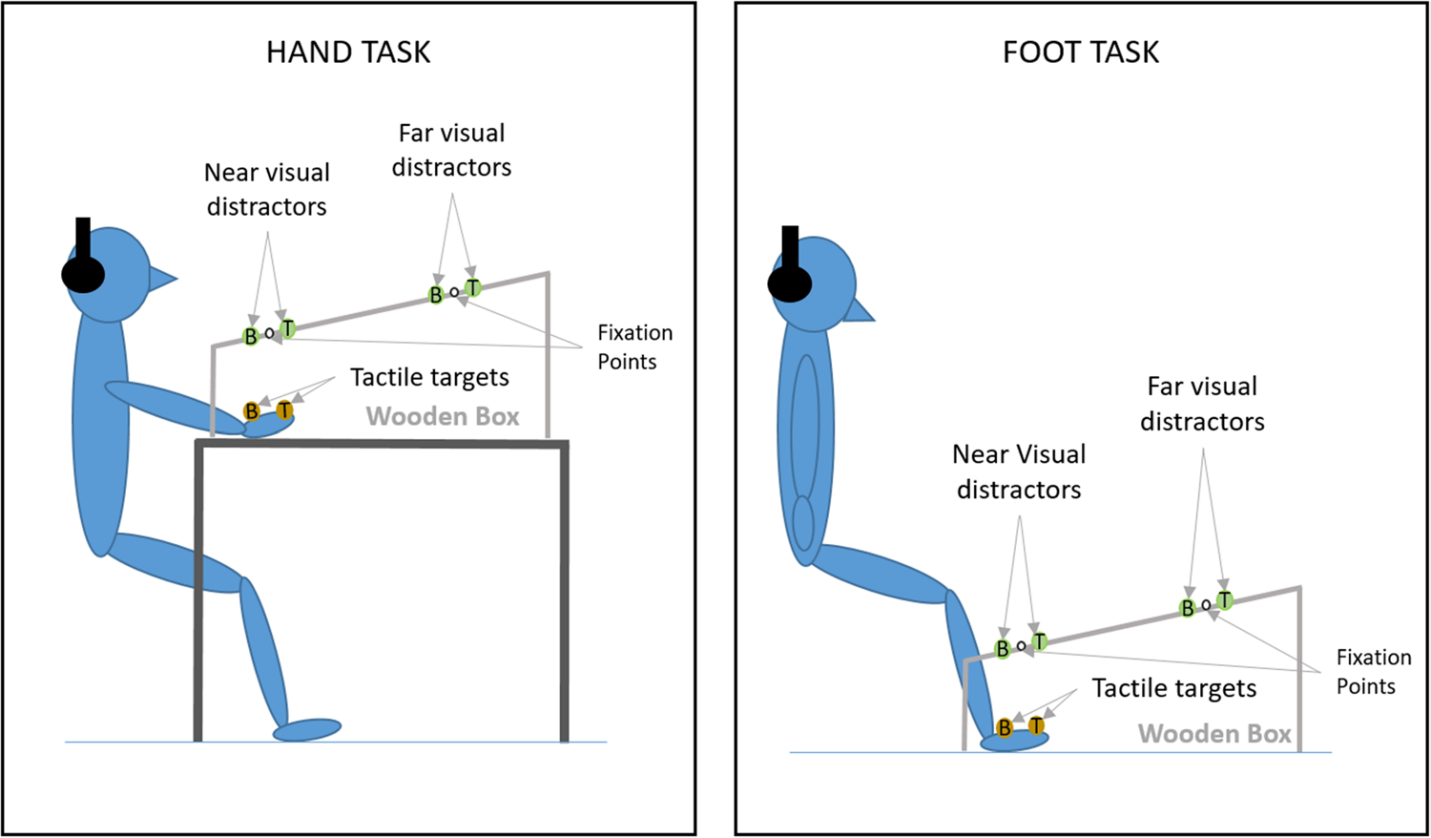
Experimental set up for the hand task (left) and the foot task (right). Yellow circles represent the tappers used to deliver the vibrotactile targets to the dorsum of the right hand or right foot, while green circles represent the LEDs used to present the visual distractors at near and far locations. The white dots represent the fixation points used for near and far distractors. The distance of the visual distractors from the touched limb (near vs. far distractors) was manipulated in different blocks of trials. Within each block, the relative location of the tactile target and of the visual distractor was selected randomly (higher vs. lower locations are labelled as ‘top’ and ‘bottom’ and indicated by the letter T and B in the figure). The tactile target and the simultaneous visual distractors were presented at the same relative location on congruent trials (both top or both bottom locations), while they were presented at different relative locations on incongruent trials (one top and the other bottom and vice-versa)

The experiments were conducted in a dimly lit room. Tactile targets were presented via one of two miniature solenoids tappers (Miniature Solenoid Tapper Controller Mk3, model MSTC3-4) attached to the skin of the dorsum of the right hand or foot with surgical tape. These 12 V solenoids propelled a blunt conical rod (surface area 28.27 mm^2^) onto the skin when current passed through them, producing suprathreshold tactile stimuli. Visual distractors were presented via one of four circular LEDs (4 mm diameter). Tactile and visual stimulus presentation was controlled by a Desktop Dell OptiPlex 745 computer (Dell Inc., Round Rock, TX) running an E-Prime 2.0 (Psychology Software Tools Inc., Sharpsburg, PA) script. A wooden box (see Fig. 1) was used to hide the tactually stimulated limb. The top panel of this box was slightly tilted, at an angle of approximately 40°. The four LEDs used to present the visual distractors were fixed to this top panel, they were vertically aligned with each other and positioned along an imaginary line parallel to the body midline. Two distractors were closer to the stimulated limb (near distractors) and were placed 5 cm and 10 cm from the edge of the panel, while the other two were further away from the limb (far distractors), positioned 35 cm and 40 cm, respectively, from the edge. At both near and far distractor distances, the two LEDs used to present the visual stimuli were placed 5 cm apart and a white pin positioned exactly in between served as fixation point. For both near and far distractors, the distractor above fixation was labelled top, while the distractor below fixation was labelled bottom (see Fig. 1). In the hand task, in which the tactile target was delivered to the right hand (touched limb: right hand), the wooden box with the LEDs was placed on the table on which participants placed their hands. In the foot task, in which the target was delivered to the right foot (touched limb: right foot), the box with LEDs was placed on the floor (see Fig. 1). In both the hand and the foot task, the two tappers were positioned at a distance of 5 cm from each other and the hidden limb was positioned in such a way that the location of the near visual distractor was spatially aligned with the visually hidden tactile targets (i.e. the tactile tappers were exactly underneath the near visual distractors, see Fig. 1). The tactile target closer to the fingers or toes was labelled top, while the one closer to the wrist or ankle was labelled bottom (see Fig. 1). Two vertically arranged rectangular switches (each 51.84 cm^2^ response surface) were used to record responses via a PST Serial Response Box Model 200a (Psychology Software Tools Inc., Sharpsburg, PA). These switches were placed on one side of the table when operated by the responding hand, and on the floor when they were operated by the responding foot. To mask any potential noise by the solenoids, participants listened to white noise through headphones during the experiment (Sony MDR-V150 Dynamic Stereo Headphones Sony Corporation, Tokyo).

### Procedure

**Fig. 2:**
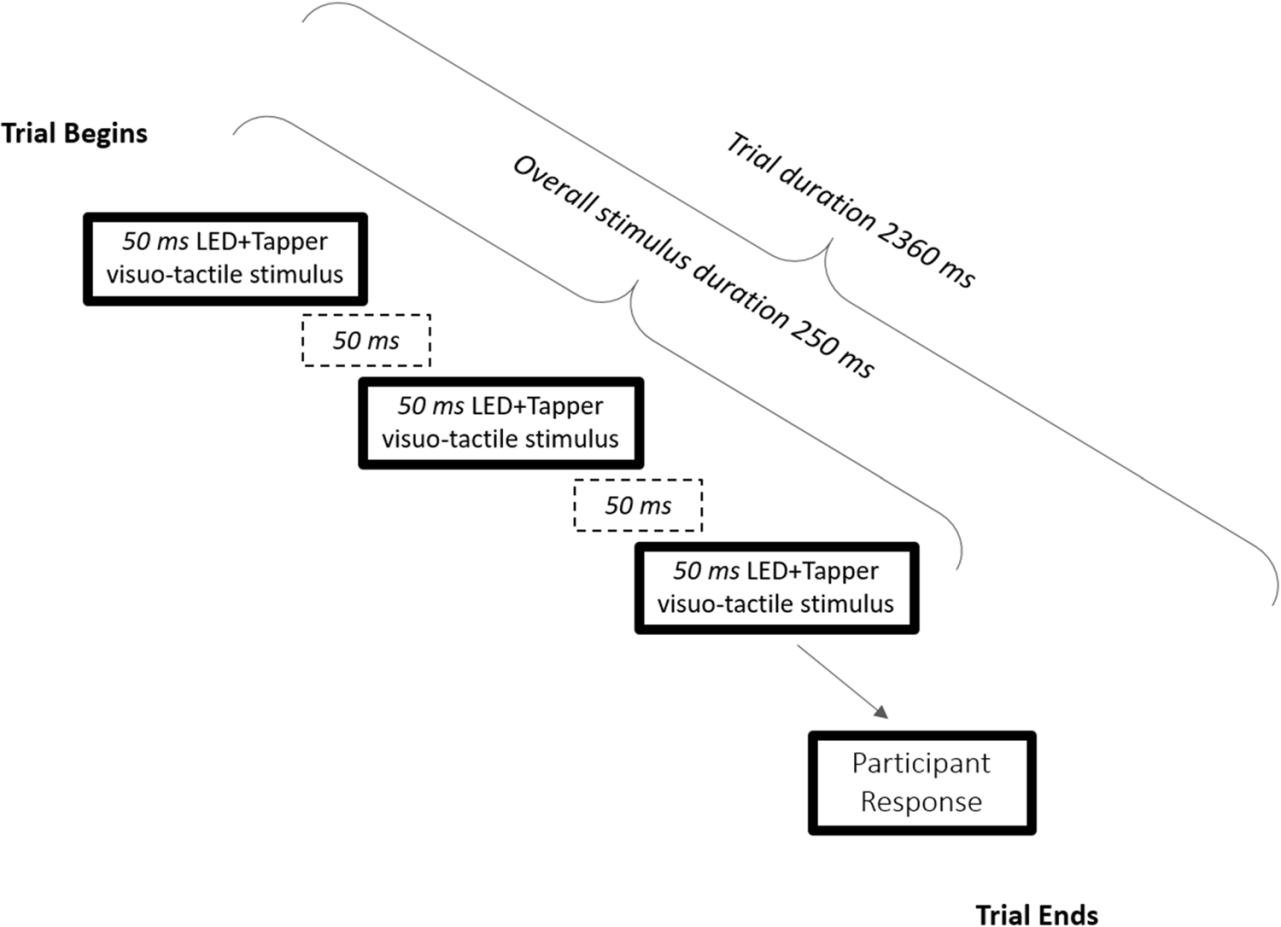
Schematic representation of the timeline of the trial events

Each trial began with the presentation of three 50 ms long pulses of one vibrotactile target and one visual distractor, separated by two 50 ms long offset periods (see Fig. 2). The onset and offset of the visual and tactile pulses were simultaneous. Overall, the duration of the visual distractor and tactile targets was 250 ms long. Following stimulus onset an interval of 2360 ms was used to record responses. At the end of this interval, the trial was terminated (see Fig. 2).

Each participant completed 16 blocks of 60 trials each (960 trials in total). The touched limb (hand task vs. foot task) and the distractor distance (near vs far distractors) were manipulated within participants in different blocks of trials. Each block consisted of 15 repetitions of the 4 possible combinations of target location (i.e. top or bottom) and distractor location (i.e. top or bottom). Thus, 30 congruent trials (the elevation of the tactile target matched the elevation of the visual distractor, both top or both bottom locations) and 30 incongruent trials (the elevation of the tactile target did not match the elevation of the visual distractor, one top and the other bottom or vice-versa) were randomly presented within each block. On four consecutive blocks, participants performed the hand task in which the tactile stimuli were delivered to the right hand. In the remaining four blocks, they completed the foot task in which the tactile stimuli were presented to the right foot. The distractor distance (near vs far) changed after 2 consecutive blocks of 60 trials each. That is, for two consecutive blocks visual distractors were presented at near locations (above the stimulated limb) while for the remaining two they appeared at far locations (35–40 cm from the limb). Task order (hand task followed by foot task or vice-versa) was counterbalanced across participants, as was the order of the distractor distance within each task (near followed by far distractors or vice-versa).

Different groups of participants performed the same visuo-tactile hand and foot tasks using different effectors to execute the responses, as mentioned above in the participants’ description. That is, the responding effector was manipulated between participants. This manipulation was aimed at investigating whether response requirements (i.e. responding with the hand or the foot) modulated the size of the visuo-tactile CCE measured in the hand and in the foot task. It is relevant to note that the limbs that received the tactile stimulation (right hand in the hand task and right foot in the foot task) were never used as responding effectors. Thirty-two participants (homologous response group) used their left hand to operate the response keys to respond to the elevation of tactile stimuli presented to the right hand (in the hand task) and their left foot to operate the response keys to respond to the elevation of tactile stimuli delivered to the right foot (in the foot task). Twenty participants (non-homologous response group) used their right foot to respond to tactile stimuli to the right hand (in the hand task) and right hand to respond to tactile stimuli presented to the right foot (in the foot task).

Upon arrival, participants completed the handedness questionnaire (Oldfield 1971). Depending on the exact task order, the tappers were then placed to their right hand or foot. Participants were then instructed as to their stimulus–response mapping and completed a 60 trials training block to ensure that they could differentiate between top and bottom stimuli. Whenever the overall accuracy was lower than 70% the training block was repeated. The training block was then repeated before the start of the second task after the tappers were placed on the new body locations. The data from these training blocks were not analysed.

Participants were instructed to respond as fast and as accurately as possible to the elevation of the tactile target (top or bottom) using the vertically arranged response top and bottom keys (top response for top targets and bottom response for bottom targets). They were also instructed to ignore the visual distractors and maintain their eyes on the relevant fixation point throughout the experimental task. A CCTV camera allowed the experimenter to monitor eye movements and eye fixation during the tasks.

### Design and analysis

A 2 (touched limb) × 2 (distractor distance) × 2 (crossmodal congruency) × 2 (responding effectors) design was used to investigate whether the crossmodal congruency effect (CCE) measured at near and far locations varied as a function of the touched limb. The variable touched limb (hand task vs. foot task) indicated the location of the tactile tappers on the body (hand vs. foot) in a given block of trials. The variable distractor distance indicated the spatial separation between the tactually stimulated body part and the visual distractors (near, distractors right above the limb, vs. far, distractors 30–35 cm distant from the touched limb). The variable crossmodal congruency coded the spatial relationship (congruent vs. incongruent) between the location of the tactile target (top or bottom, regardless of which limb received the stimulation) and the irrelevant distractor (top or bottom, regardless of body-distractor distance). The variables touched limb, distractor distance and crossmodal congruency were manipulated within participants. Finally, the variable responding effectors (homologous vs. non-homologous responses) indicated the effectors used to respond to the elevation of tactile stimuli in the different tasks and was manipulated between participants (see Table 1). For one group of participants (the homologous response group), responses were executed with the left hand in the hand task and with the left foot in the foot task (while their right hand and right foot received the tactile stimulation, respectively), whereas the other group (the non-homologous response group) used the right foot in the hand task and the right hand in the foot task (while their right hand and right foot received the tactile stimulation, respectively). Mean RTs and accuracy rates were submitted to separate mixed analyses of variance (ANOVAs) with touched limb (hand task vs. foot task), distractor distance (near vs. far distractors) and crossmodal congruency (congruent vs. incongruent) as within-subjects factors and responding effectors (Homologous vs. Non-homologous response) as between-subjects variable. Only correct responses were included in the RT analyses. The assumption of Homogeneity of variances between the groups was tested using the Levene’s test, while normality was tested with the Shapiro–Wilk normality test. Accuracy and RT data were entered in the ANOVAs only after the Levene’s test revealed that the homogeneity assumption was met (*p* > 0.05). Shapiro–Wilk normality test showed that residuals were normally distributed (*W* = 0.99, *p* > 0.06).

**Table 1:** Summary of the experimental design and main results reported separately for each group

Between-subject factor	Responding effectors		Homologous response group				Non-homologous response group				All participants				
			Left hand		Left foot		Right foot		Right hand						
Within-subjects factors	Touched limb (Task)		Hand task Right hand		Foot task Right foot		Hand task Right hand		Foot task Right foot		Hand task Right hand			Foot task Right foot	
	Distractor distance		Near	Far	Near	Far	Near	Far	Near	Far	Near		Far	Near	Far
	Crossmodal Congruence	Congruent	641	651	682	691	614	608	700	689	627		630	691	690
		Incongruent	724	720	754	768	708	679	778	784	716		699	766	776
		CCE	84*	68*	71*	78*	94*	71*	78*	95*	89*		69*	75*	86*

Asterisks reflect significant CCEs, *p* < 0.05

## Results

Participants failed to respond on 4.6% of all trials while they made a choice error (responded incorrectly) on 2.7%^2^ of all trials. As such, 7.3% of all trials in total were removed from the RT analysis.

The mixed ANOVA carried out on accuracy rates revealed a main effect of touched limb (*F*(1,50) = 7, *p* = 0.011, ${\eta }_{p}^{2}$= 0.12) showing that elevation responses were more accurate when tactile stimuli were delivered to the hand (93.5%) than to the foot (91.6%). As expected, we also found a main effect of crossmodal congruency, with responses more accurate for congruent than for incongruent visuo-tactile trials (95% and 90%, respectively; *F*(1,50) = 52.9, *p* < 0.001, ${\eta }_{p}^{2}$= 0.5). Finally, a significant touched limb by crossmodal congruency interaction revealed that the congruency effect was larger for feet (6% CCE; 94.6% congruent and 88.6% incongruent) than for hands (3.7% CCE; 95.3% congruent and 91.6% incongruent). No other main effects or interactions were significant [distractor distance, *F*(1,50) = 0.57, *p* = 0.45, ${\eta }_{p}^{2}$= 0.01; responding effectors, *F*(1,50) = 0.16, *p* = 0.68, ${\eta }_{p}^{2}$= 0.003; touched limb × responding effector, *F*(1,50) = 0.9, *p* = 0.3, ${\eta }_{p}^{2}$= 0.019; distractor distance × responding effectors, *F*(1,50) = 3, *p* = 0.088, ${\eta }_{p}^{2}$= 0.057; crossmodal × responding effectors, *F*(1,50) = 0.001, *p* = 0.9, ${\eta }_{p}^{2}$= 0.001; touched limb × distractor distance, *F*(1,50) = 0.15, *p* = 0.7, ${\eta }_{p}^{2}$= 0.003; distractor distance × crossmodal congruency, *F*(1,50) = 0.73, *p* = 0.4, ${\eta }_{p}^{2}$= 0.014; touched limb × distractor distance × responding effectors, *F*(1,50) = 1.3, *p* = 0.26, ${\eta }_{p}^{2}$= 0.025; touched limb × crossmodal congruency × responding effectors, *F*(1,50) = 0.4, *p* = 0.5, ${\eta }_{p}^{2}$= 0.008; distractor distance × crossmodal congruency × responding effectors, *F*(1,50) = 0.76, *p* = 0.39, ${\eta }_{p}^{2}$= 0.015; touched limb x distractor distance × crossmodal congruency, *F*(1,50) = 1.4, *p* = 0.24, ${\eta }_{p}^{2}$= 0.027; touched limb × distractor distance × crossmodal congruency × responding effectors, *F*(1,50) = 0.19, *p* = 0.66, ${\eta }_{p}^{2}$= 0.004].

The ANOVA carried out on mean RTs revealed a significant main effect of touched limb [*F*(1,50) = 20.1, *p* < 0.001, ${\eta }_{p}^{2}$= 0.29]. Responses were generally faster when tactile targets were presented to the hand as compared to the foot (672 ms and 729 ms, respectively). Responses to congruent visuo-tactile stimuli were faster than responses to incongruent ones (661 ms and 740 ms, respectively), as demonstrated by the main effect of crossmodal congruency [*F*(1,50) = 313, *p* < 0.001, ${\eta }_{p}^{2}$= 0.86]. There was no main effect of distractor distance [*F*(1,50) = 0.05, *p* = 0.85, ${\eta }_{p}^{2}$= 0.001] nor significant interactions between touched limb and distractor distance [*F*(1,50) = 0.74, *p* = 0.39, ${\eta }_{p}^{2}$= 0.015], or distractor distance and crossmodal congruency [*F*(1,50) = 0.6, *p* = 0.4, ${\eta }_{p}^{2}$= 0.013]. In addition, there was no significant interaction between touched limb and crossmodal congruency [*F*(1,50) = 0.07, *p* = 0.78, ${\eta }_{p}^{2}$= 0.002]. Despite the fact that tactile stimuli were presented to body parts with different spatial acuity and that visual stimuli in the hand and foot task were characterised by different distances from the eyes, the interference of the visual distractor on the tactile target was similar when visuo-tactile stimuli were presented near/to the hand and near/to the foot (81 ms CCE in the foot task and 80 ms CCE in the hand task).

**Fig. 3:**
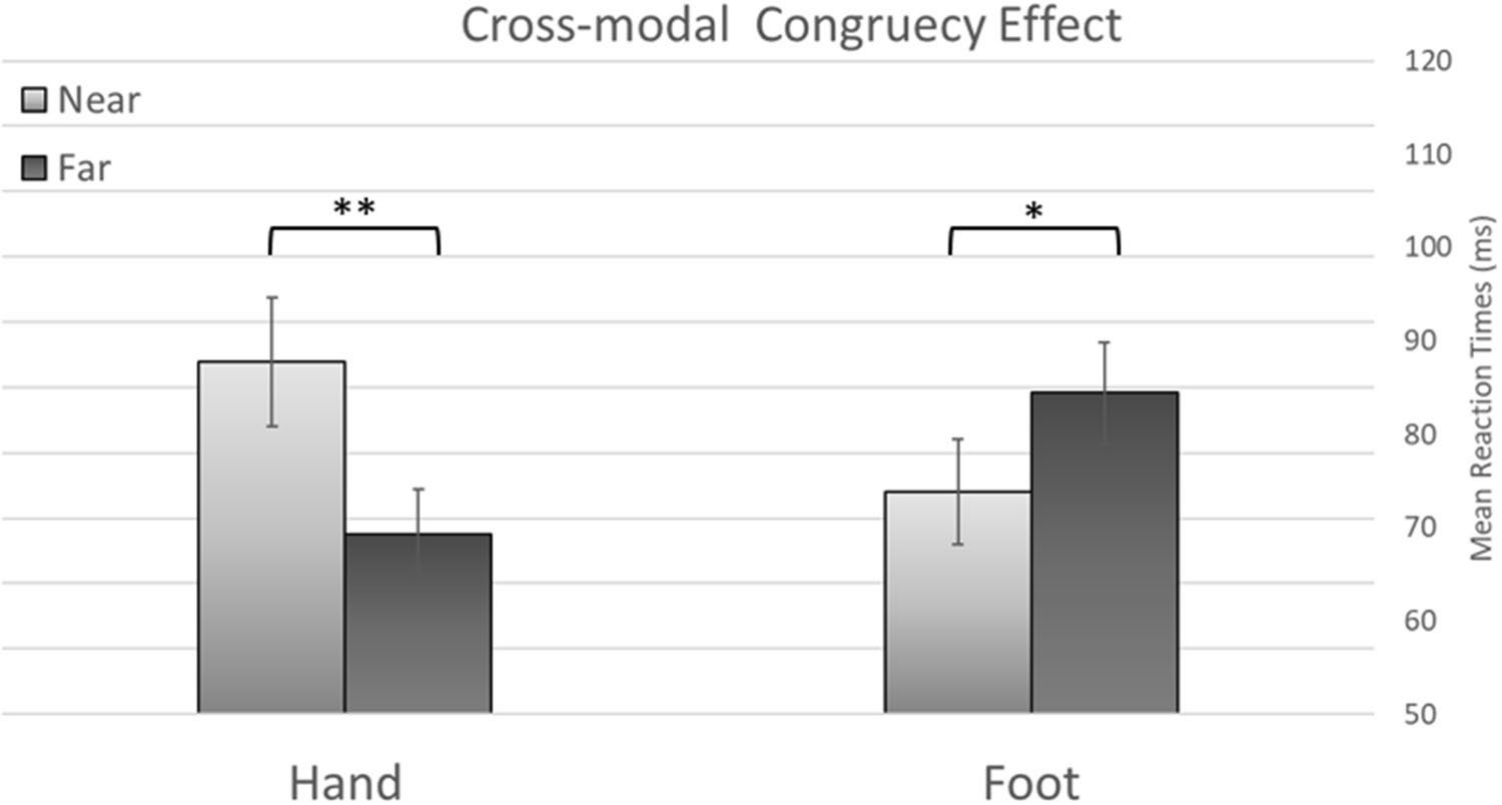
The CCE measured in the hand task (left) and foot task (right), shown separately for near visual distractors (light grey) and far visual distractors (dark grey). Error bars represent the standard error of the means. The asterisks indicate the significance level of the pairwise contrasts: **p < .01; *p < .05

The RT analysis also revealed a significant three-way interaction between touched limb, distractor distance and crossmodal congruency [*F*(1,50) = 13.37, *p* < 0.001, ${\eta }_{p}^{2}$= 0.21], see Fig. 3. Because the aim of this study was to evaluate possible differences in near and far space between the CCE observed in the hand and in the foot tasks, follow-up ANOVAs were carried out separately for these tasks. In the hand task, when the tactile target was presented to the hand, a significant distractor distance by crossmodal congruency interaction was observed [*F*(1,50) = 7.8, *p* < 0.007, ${\eta }_{p}^{2}$= 0.13]. This demonstrates that the crossmodal congruency effect observed at near distractor locations [difference between congruent and incongruent trials, 627 ms and 716 ms, respectively, 89 ms CCE; *t*(51) = 14.7, *p* < 0.001] was larger than that observed at far locations [congruent, 630 ms and incongruent, 699 ms; 69 ms CCE; *t*(51) = 12.6, *p* < 0.001]. In the foot task, a significant distractor distance by crossmodal congruency interaction was also observed [*F*(1,50) = 4.3, *p* = 0.043, ${\eta }_{p}^{2}$= 0.08]. However, in the foot task, the crossmodal congruency effect observed at near distractor location (difference between congruent and incongruent trials, 691 ms and 766 ms, respectively, 75 ms CCE; *t*(51) = 13, *p* < 0.001) was smaller than that observed at far locations [congruent, 690 ms and incongruent, 776 ms; 86 ms CCE; *t*(51) = 15.6, *p* < 0.001].

The between-subjects factor responding effectors was not significant [*F*(1,50) = 0.09, *p* = 0.7, ${\eta }_{p}^{2}$= 0.002] and did not interact with any of the other factors [all *F*(1,50) < 2, all *p* > 0.1, all ${\eta }_{p}^{2}$< 0.05]. Crucially, the fact that the interaction of interest touched limb × crossmodal congruency × distractor distance was not further modulated by responding effector [*F*(1,50) = 1.2, *p* = 0.27, ${\eta }_{p}^{2}$= 0.023] indicates that responding with the homologous or non-homologous effector with respect to the tactually stimulated limb did not change the pattern of the visuo-tactile CCEs observed in the hand and foot task. This is further supported by the fact that the interaction of interest touched limb × crossmodal congruency × distractor distance was significantly present in both participants groups (homologous response group: *F*(1, 31) = 4.8, *p* = 0.036, ${\eta }_{p}^{2}$ = 0.13; non-homologous response group: *F*(1, 19) = 7.7, *p* = 0.012, ${\eta }_{p}^{2}$ = 0.29). See Table 1 for a summary of the data reported separately by group.^3^

## Discussion

In this study, we investigated the spatial properties of the representation of PPS around the upper and the lower limbs (hands and feet). We measured the crossmodal congruency effect (CCE) as an index of the strength of visuo-tactile integration, while we manipulated the distance in depth between a tactile target delivered to the hand (hand task) or the foot (foot task) and a simultaneous task-irrelevant visual distractor presented in near or far space. Results showed the presence of reliable CCEs in both the hand and the foot tasks, providing additional evidence for the presence of PPS representations around both the hands and feet (Schicke et al. 2009; Van Elk et al. 2013). Crucially, however, we observed systematic differences between the spatial properties of the CCE in near vs. far space observed for the hand and foot.

In the hand task, in which the tactile target was delivered to the back of the hand, visuo-tactile integration was stronger in near than in far space. In line with existing evidence, the CCE reflecting hand PPS decreases as the tactile target—visual distractor distance increases (e.g. Holmes et al. 2007; Holmes 2012). This pattern has been observed not only when the visual distractor was presented far from the tactually stimulated hand and close to the opposite non-stimulated hand (e.g. Pavani et al. 2000; Spence et al. 2004a, b; Soto-Faraco et al. 2004), but also when it was presented further away from the body closer to the boundary separating peripersonal space from extrapersonal space (e.g. Holmes 2012). This body of evidence has often been interpreted as evidence for a PPS in humans with similar properties to those reported in neurophysiological studies in non-human primates (e.g. Graziano et al. 1997; Iriki et al. 1996).

Intriguingly, an opposite pattern of results characterised the foot PPS. In the foot task in which the tactile target was presented to the back of the foot, a stronger interference effects was created by the visual distractors when they were delivered further away from the foot (far space) as compared to when they were closer to it (near space). This finding provides the first direct evidence that the foot PPS is characterised by different spatial properties as compared to the hand PPS. One previous study using a visuo-tactile task with approaching or receding visual stimuli suggested that the boundary of foot PPS was located approximately 70 cm away from the foot (Stone et al. 2018). Other studies using a similar audio-tactile and visuo-tactile task with approaching and receding stimuli have revealed that the boundaries of hand PPS were located approximately 30–50 cm from the hand (e.g. Serino et al. 2015). Although these observations come from different studies (hand PPS and foot PPS in near and far space have not been directly contrasted directly), together they suggest the presence of a larger PPS around the lower limbs as compared to the upper limbs one. Our results complement and expand this body of evidence showing that the foot PPS is stronger in far space than in near space, at a distance where the hand PPS has already started to decrease. This demonstrates that distinct PPS representations centred on different limbs are characterised by distinct spatial properties.

Differences between hand and feet PPS representations in the literature primarily concern the amount of plasticity (or lack thereof) of the foot PPS as compared to the hand PPS. For example, comparable hand PPS were observed during a CCT in which the stimulated hands were visible as well as when two rubber hands were shown instead (e.g. Pavani et al. 2000). However, when the rubber hands were shown in an anatomically implausible position (a fake left hand positioned where the hidden real right hand was placed and vice-versa) the CCE disappeared (e.g. Pavani et al. 2000). In contrast, when the foot PPS was measured the impact of viewing fake limbs in anatomically impossible/incongruent positions had less dramatic effects on the CCE (Van Elk et al. 2013). That is, the hand PPS but not the foot PPS was dynamically updated (see also Pozeg et al. 2015).

Together, the facts that hand and foot PPS have different spatial properties and show different levels of plasticity suggest the presence of independent PPS representations for the different limbs. One of the primary functions of PPS is to allow the successful implementation of goal-directed movements (e.g. Rizzolatti et al. 1981; 1997). Accordingly, PPS can be seen as a multisensory interface that allow motor interactions between the body and its environment. This can be achieved thanks to the flexible nature of the PPS representations which can be quickly updated to reflect changes in the covert planning of participants’ actions (Brozzoli et al. 2009; 2010). A close link between action and PPS has been shown for the hands whereby visuo-tactile interactions in far space become stronger after participants practice goal-directed movements with long tools allowing them to interact with objects in extrapersonal space, essentially extending their reachable space (e.g. Holmes et al. 2007; Holmes 2012; Serino et al. 2015). Furthermore, the hand PPS can be plastically modulated also by the simple planning and execution of goal-directed grasping movements (Brozzoli et al. 2009; 2010). It was suggested that movement planning leads to a body part specific remapping of PPS (Brozzoli et al. 2009; 2010; Patane et al. 2019).

Given the close link between action and PPS we suggest that the different spatial properties observed for hand and foot PPSs in the present study are due to the different functional roles of upper and lower limbs. The upper limbs are involved in a variety of different movements, and hand goal-directed actions often involve interactions with objects. On the other hand, locomotion is one of the primary function of lower limbs, although they can also be involved in interactions with objects (e.g. kicking a ball). It is conceivable that different sector of space have selective functional properties for hand and foot PPSs. For example, given the dynamic properties of locomotion, ‘far’ space can be considered more relevant than ‘near’ space within the foot PPS, because foot actions (walking, kicking a ball, etc.) tends to involve an active movement of the whole body towards the edge of PPS. Detecting sudden obstacles in far space could leave sufficient time to adjust and adapt the movement planning accordingly, whereas prioritizing the representations of objects/obstacles in near space could lead to an inability to avoid the obstacle. Interestingly, it has been suggested by Stone et al. (2018) that the putative boundary observed for the foot PPS coincided approximately with the average step length (e.g. Sekiya et al. 1997). An indirect link between PPS representation around the lower limbs and movement has been demonstrated in a recent study investigating the feet PPS of individuals with spinal cord lesions (Scandola et al. 2016). Although PPS around the lower limbs of paraplegic individuals was reduced as compared to control participants to the point that no reliable CCE was observed, the PPS representations around the feet were partially restored when the limbs were passively moved before the CCT (Scandola et al. 2016). These findings are important, because they not only confirm the functional link between possible feet actions and feet PPS presence/extension (PPS absence when movements are not possible), but they also highlight the dynamic properties of the feet PPS which appears to be constantly updated, similarly to what observed for the hand PPS. More recently, a dynamic remapping of the space around the body has also been shown during walking (Noel et al. 2015). Using an audio-tactile interaction task as a proxy for the PPS boundary, results suggested that PPS boundaries extend while walking as compared to a still condition. Intriguingly, a similar shift of PPS boundary was also observed in the absence of an actual walking movement, when a walking movement sensation was induced through the presentation of a walking-sound vibration under the soles of participants feet (Amemiya et al. 2019). Together, these findings show that movement of the feet, and more specifically walking, can dynamically induce a remapping of the space around the body.^4^

At first, our finding that the CCE around the lower limb is stronger at far locations as compared to near feet locations may seem in contrast with existing evidence. For example, Stone and colleagues using a different visuo-tactile paradigm observed that responses to the tactile target presented to the foot were faster when an approaching visual distractor reached locations that were nearer the body as compared to the ones that were further away from it (Stone et al. 2018). This suggests that the boundary of foot PPS is located between near and far locations, where response times start to increase (approximately 70 cm from the body). However, in that study, the space immediately above the foot was not tested (‘near’ locations were those 22 cm away from the foot). Our results complement and expand these existing findings. Together they show that the representation of space around the foot is weaker in the space immediately above the foot, increases between 20 and 45 cm from the foot, and drops again as PPS boundary is reached (between 45 and 90 cm from the foot).

Importantly, different paradigms assess different aspects of PPS. The visuo-tactile task used by Stone and colleagues (and adapted from the audio-tactile task first introduced by Canzoneri et al. 2012) is particularly suited to detect the boundary of PPS thanks to the use of dynamic stimuli which allow to measure PPS along a continuum between near and far space. In this task, the dependent variable of interest is the speed of RTs to tactile targets as a function of distance from the dynamic distractor. In contrast, the CCT allows to quantify the strength of visuo-tactile integration at specific discrete locations in space assessing the size of the CCE. Using the CCE as a proxy for PPS allows for the direct comparisons of the strength of visuo-tactile integration measured at different locations and for the different effectors. Thus, exploring the same phenomenon using different tasks is crucial, because they offer insights into complementary aspects of PPS.

Finally, it is worth noting that the responding effectors did not modulate the differences observed between hand and foot PPS. This observation is relevant, because previous studies have shown that the specific effector used to respond in the CCT plays a relevant role in determining the strength of the visuo-tactile integration (Gallace et al. 2008). In the present study, we asked different groups of participants to respond to the elevation of the tactile target by pressing a top or bottom response key using the homologous effector contralateral to the touched limb (left hand in the Hand task, and left foot in the Foot task) or the non-homologous effector ipsilateral to the touched limb (right foot in the Hand task and right hand in the Foot task) to rule out the possibility that the responding effector may impact the representation of PPS measured around the hand and foot. Furthermore, in all experimental conditions, all limbs (responding effectors as well as touched limbs with respect to which PPS was measured) were hidden from view, because existing evidence has demonstrated that visual perception is biased around the hands (e.g. Abrams et al. 2008; Reed et al. 2006) and feet (Stettler and Thomas 2016). Results were characterized by a similar pattern across different responding effectors groups, suggesting that although different responding effectors are likely to impact the coding of space (c.f. Gallace et al. 2008) the differences observed between foot and hand PPS in near and far space were not driven by it.

In conclusion, the present study confirms the presence of a multisensory representation of PPS not only around the hand but also around the feet. Crucially, while the strength of the integration of visuo-tactile information around the upper limbs decreases as visual distractors are presented further away from the hand (in depth), we show that multisensory integration around the lower limbs increases as the distance from the body increases. Taking into account our findings and previous evidence, this difference could be due to the functional role of upper and lower limbs.

## Data Availability

The datasets generated during and/or analysed during the current study are available from the corresponding author upon request.

## Footnotes

1 It should be noted that we only assessed hand preference in the present study. This was driven by the need to control for hand dominance given its clear impact on PPS tasks, as demonstrated by Hobeika et al. (2018). Future studies could also assess footedness, for example using the WFQ (Elias et al. 1998), to document any effect of foot preference on PPS since at present there is no clear evidence.

2 It is worth noting that the number of choice errors was very low (2.7% of all trials). This, together with a lack of significance in the error analysis for the interaction of interest (i.e. touched limb × crossmodal congruency × distractor distance, *F*(1,50) = 0.19, *p* = 0.66, ${\eta }_{p}^{2}$= 0.004) suggest that errors as a dependent variable were not a sensitive enough measure to uncover spatial modulations of the CCE in the present study. As such we focussed the discussion on the more sensitive RTs.

3 Because the factor responding effector was manipulated between participants, but the two groups of participants had unequal sizes, we also analysed the same RT data using a linear mixed model (LMM) analysis to rule out the possibility that the results of the ANOVA were affected by the unequal samples. The effects of the fixed factors responding effector (group), touched limb, distractor distance and crossmodal congruency were tested using trial level data as dependent variable and participants as random intercept. The model with all the factors was a better predictor than the model with only three fixed factors (AIC = 610143, logLik = − 305054, × 2(8) = 208, *p* < 0.001). Crucially, the interaction of interest between touched limb, distractor distance and crossmodal congruency was significant (F 1,46058 = 19.77, *p* < 0.001) while the between-subjects factor responding effector did not further modulate this interaction (F 1,46058 < 3.0637, *p* > 0.05). Thus, results are virtually identical to those reported in the ANOVA, suggesting that in this instance the presence of unequal samples did not impact results. This is further confirmed by the analyses carried out separately for each group showing significant interactions of interest between touched limb × distractor distance × crossmodal congruence in each group.

4 Serino and colleagues (Serino et al. 2015) have shown the existence of independent PPS representations centred on distinct body parts such as the trunk, the hand, and the face. Because in both Noel et al. (2015) and Amemiya et al. (2019) studies, participants were asked to respond to a tactile stimulus presented to the chest (while a looming or receding sound was presented), it is unclear what is the contribution of the different body parts (specifically the contribution of the feet) to the presence and characteristics of the observed PPS. Future studies should specifically address this by presenting the tactile stimuli directly to the feet.

## Code availability

Commercial software applications were used to present the stimuli during the experiment and analyse the data. These are listed in the manuscript.
